# Assessment of the Awareness and Use of Quality of Life Tools in Small Animal Practices in Germany

**DOI:** 10.3390/ani15243617

**Published:** 2025-12-16

**Authors:** Friederike Felicitas Rhein, Rebecca Klee, Balazs Albrecht, Stephanie Krämer

**Affiliations:** 1 Professorship of Laboratory Animal Science and Animal Welfare & Interdisciplinary Centre for Animal Welfare Research 3R (ICAR3R), Justus Liebig University Giessen, Frankfurter Str. 95, 35392 Giessen, Germany; stephanie.kraemer@vetmed.uni-giessen.de; 2 Boehringer Ingelheim Vetmedica GmbH, Binger Straße 173, 55216 Ingelheim am Rhein, Germany; rebecca.klee@boehringer-ingelheim.com (R.K.); balazs.albrecht@boehringer-ingelheim.com (B.A.)

**Keywords:** quality of life, companion animals, dog, cat, assessment, instrument, tool

## Abstract

The assessment of animal welfare and quality of life (QoL) is a critical aspect of veterinary practice. While the development of standardized tools to measure QoL has been widely discussed, there is limited research analyzing how these tools are applied in clinical practice. This study investigates the current state of QoL assessment in small animal medicine in Germany through a mixed-methods approach. A survey was conducted with veterinarians and veterinary assistants, assessing the awareness and use of QoL assessment tools in practice. In addition, interviews with veterinarians were conducted to examine their perceptions and experiences regarding the use of QoL measurement tools and the assessment of QoL itself. The findings indicate that the use of standardized QoL assessments is rare and often primarily done while doing studies. Factors hindering the widespread use of these instruments include lack of time, lack of awareness, the perceived lack of need and resistance to fitting the individuality and subjectivity of QoL into a schema. As beneficial features of such tools, veterinarians mentioned the possibility of documenting and tracking QoL and the enhancement of the relationship between professionals and clients. Further research should therefore not only focus on development but also on dissemination of such tools.

## Simple Summary

Quality of life (QoL) plays an important role in veterinary medicine. Over the last decades, different tools have been developed to assess the QoL of an animal in a more structured way. While there is a lot of research on how to develop and improve such tools, there is little knowledge about whether and to what extent those tools are used in veterinary practice. This study combined an online survey and expert interviews to investigate the awareness and usage of QoL assessment tools in small animal practices. Results show that only a minority of veterinarians are aware of the existence of such tools and that there is little to no use for them in the daily veterinary routine of small animal medicine practitioners in Germany. Besides the lack of awareness, reasons for not using such tools on a regular basis were found to be lack of time, perceived lack of need and resistance to fitting the individuality and subjectivity of QoL into a schema. At the same time positive attitudes toward such assessments were found. Veterinarians especially highlighted the potential of such tools to improve communication with the owners and the majority reported a willingness to use them.

## 1. Introduction

Assessing quality of life (QoL) of an animal is a core competence of a veterinarian and guides decisions regarding treatment or euthanasia [1]. Due to animals’ inability to self-report, their QoL must be assessed via proxies, such as the owner, the treating veterinarian or a veterinary technician. To quantify such assessments there have been several attempts to develop instruments to measure QoL [2,3,4,5], thus making it comparable, for example, by evaluating the state of an animal pre- and post- intervention. Particularly in patients with chronic diseases, where changes in the condition may be low and therefore remain undetected, a standardized assessment of QoL can be beneficial [1].

Tools to measure QoL can take unstructured forms, such as a single scale rating, or structured forms, such as a questionnaire [3,6].

Similarly to human medicine, instruments to assess QoL in small animal veterinary medicine also appear as generic or disease-specific instruments [4,5]. Generic instruments are designed to capture the overall state of an animal. While these instruments can detect changes in areas not primarily related to the disease and thus capture unexpected side effects of a therapy, they may be less sensitive to smaller, more specific changes in certain organ systems due to their broad scope [6]. Disease-specific instruments, on the other hand, can ask more detailed questions about relevant areas but may miss changes in unexpected areas [6]. Disease-specific instruments to assess QoL in small animals have been developed for various fields, including orthopedic and neurological conditions, as well as internal medicine such as cardiological, endocrinological, or dermatological diseases [3,5].

Though the existing instruments often lack a thorough evaluation of their psychometric properties, their usage can, if done carefully, subject to the restrictions, still be beneficial in daily practice. They can serve as a guideline and promote discussions between owners and veterinarians about their pet’s QoL [7].

Pet owners also mostly consider the QoL of their animals to be more important than lifespan and are willing to trade up to six months of their pet’s life span for an improved QoL [8]. Additionally, initial studies indicate that pet owners have a positive attitude towards the use of questionnaires to assess QoL in primary care settings and find them enriching [9]. Pet owners approve discussions about their pet’s QoL and well-being with their veterinarian and are interested in completing a questionnaire to evaluate their pet’s QoL in their own time [10].

Completing a health-related QoL questionnaire can additionally lead to positive client communication experiences for pet owners and can enhance their perception of the overall service [9]. It further enables more profound conversations about QoL between professionals and owners [9] and may initiate discussions about QoL during routine check-ups, thus increasing the effectiveness of such consultations [7].

It is undeniably important that the quality of instruments for measuring QoL continues to improve. However, whether this ultimately results in an improvement for patients in everyday settings also depends crucially on whether such questionnaires are used at all in everyday veterinary practice.

A survey-based study investigated the awareness and use of QoL tools in the UK and found that less than four percent use such tools in daily practice [11].

As general research communication mostly occurs in English, most developed instruments are also published in English. This circumstance may be a limitation for the awareness of such instruments in non-English-speaking countries, such as Germany.

In case there is an awareness of such tools, a tool would need to be translated before use in a non-English speaking country. Moreover, not only is the translation of a psychometric instrument a difficult task, because a translated instrument also needs to be validated in the new language [12], but the translation process would also take time. With time being a huge limiting factor in daily veterinary practice [13] the necessary translation before the possible use of an instrument may be an additional obstacle to their use.

This study aimed to investigate the awareness and use of QoL instruments and the current state of QoL assessment in veterinary small animal medicine in Germany, using a mixed-methods approach.

## 2. Materials and Methods

The study of awareness and use of QoL tools was part of a larger investigation concerning QoL. It encompassed the combination of a survey containing quantitative and qualitative elements, by including closed-ended and open-ended questions, and qualitative, semi-structured interviews. The combination of quantitative and qualitative research elements is referred to as mixed methods and aims to provide a more comprehensive understanding of a research problem, compensating for the respective disadvantages of each method (lack of depth in quantitative data, lack of sample size in qualitative interviews) [14]. The present study followed a convergent mixed-methods design. Although data collection was conducted sequentially due to organizational reasons, the survey and interview data were analyzed in parallel and independently.

### 2.1. Survey

The target groups of the survey were veterinarians and veterinary assistants practicing small animal medicine in Germany. ‘Veterinary assistants’ encompassed professionals who have completed formal vocational training as either a veterinary technician or an animal caretaker and work in the field of small animal medicine. Furthermore, owners of dogs and cats were another target group, but their data served to answer research questions that will be presented elsewhere and would be beyond the scope of this article. The survey contained 54 questions, from which up to 30 were presented to the veterinarians and up to 28 were presented to the veterinary assistants (some questions were additional depending on answers to previous questions). The remaining questions were presented to owners of dogs and cats (data will be published elsewhere). The questions discussed in the present study encompassed demographic questions regarding the gender (female, male, diverse) and age (18–30, 31–60, >60) of the participants, and how long they have been practicing in small animal medicine (<5 years, 5–10 years, 10–20 years, >20 years). The veterinarians were furthermore asked from which university they graduated and whether they have an additional qualification, such as being a Diplomate of the European College or American Board or having an equal national qualification, to characterize the study sample.

The participants were then asked whether they knew any tools to assess QoL in companion animals. If the answer was “yes” or “I am not sure”, an open-ended follow-up question was presented, asking them to describe which tools they knew, or what thoughts they were unsure about. They were then presented with information about possible ways to measure QoL, for example, with a simple scale or a questionnaire-based instrument and were asked if they measure QoL on a regular basis. Again, if the answer was “yes” or “I am not sure”, an open-ended follow-up question was presented to gather more specific information. Finally, the participants were asked whether they could imagine using a validated questionnaire to measure QoL on a regular basis. The questions regarding the awareness and use of QoL tools and their sequence can be found in the supplementary material (Figure S1).

The survey was available from November 2020 to March 2021 and was distributed via the German veterinary chambers and advertised in the “Deutsches Tierärzteblatt”, a magazine sent to every veterinarian in Germany, respondents were therefore self-selected. The survey was conducted using LimeSurvey Version 2.73.1 (LimeSurvey GmbH, Hamburg, Germany) and upon its conclusion, the data were analyzed with IBM SPSS Statistics Version 29.0.2.0 (20) (IMB Statistics GmbH, Böblingen, Germany). As the current manuscript only presents a subset of the larger study, the data included in this manuscript were solely analyzed descriptively. Inferential statistical analyses were performed for other parts of the study (to be published elsewhere).

During data cleansing, incomplete and inconsistent data sets were removed. The open-ended questions were analyzed manually to determine whether participants had indeed described a tool or a valid application of one. A tool in this context was defined as the reference to a “scale”, “score” or “questionnaire”. Responses that did not refer to a tool were not systematically analyzed; mentioned approaches were only extracted as examples.

### 2.2. Interviews

To gain a deeper understanding of QoL assessment in daily veterinary practice, the survey was complemented by additional qualitative, semi-structured, guideline-based expert interviews. The questions from the interview guidelines that are relevant to this paper can be found in the supplementary material (Table S1). The interviews were conducted from June 2021 to November 2021 with specialists from various fields of small animal medicine, to obtain the perspectives of highly qualified and experienced practitioners. Persons were considered experts when they had a certified specialization (either board-certified of the European College or American Board or accredited by an official veterinary council in Germany) and several years of experience in their respective field.

The sample size estimate was based on previously published recommendations, which showed that studies with a narrow research objective and a homogeneous sample typically achieve data saturation between 10 and 20 interviews for content analysis [15,16,17,18]. Since no group comparisons were planned and the group of respondents was considered to be fairly homogeneous due to their comparable veterinary training and additional qualifications, a sample size of 15 respondents was targeted.

Potential interviewees were identified through a targeted Google search, focusing on specialists working at German university veterinary hospitals, larger private clinics and specialized practices.

They were contacted via mail during several rounds of recruitment and were informed broadly about the interview’s topic. Specific questions were not disclosed to avoid preparation of prefabricated answers. In total, 56 potential interviewees were contacted, of whom 16 agreed to participate; their areas of specialty are displayed in Table 1. All 16 interviews were conducted, with the option of further recruitment should data saturation not be achieved. As the final interviews did not reveal any new aspects, no further recruitment was carried out. Interviews were conducted either face-to-face, via video call or via telephone, and were audio recorded.

The recordings were later transcribed and analyzed via a qualitative content analysis by Kuckartz and Rädiker [19] using MAXQDA Analytics Pro 24.10.0 (VERBI GmbH, Berlin, Germany). Qualitative content analysis enables the structured analysis of textual data.

The main categories can be found in Table 2 and were developed in accordance with the following research questions: (1) To what extent are small animal medicine specialists aware of standardized tools for QoL assessment? (2) Do small animal medicine specialists use standardized tools for QoL assessment, or could they imagine doing so? (3) What are the arguments for and against their use? (4) How do small animal medicine specialists assess the QoL of their patients in their daily veterinary practice?

The interview transcripts were searched for passages in which the interviewees provided information on aspects relevant to one of the main categories, and these passages were labeled with the corresponding main category.

Following this, in a second coding cycle, the text passages assigned to each of those main categories were analyzed one by one. For each aspect that was found, either a new category was created or—particularly as the process progresses—was assigned to one of the existing categories. In this way, inductive subcategories were developed for each main category to characterize the content.

## 3. Results

### 3.1. Survey

#### 3.1.1. Veterinarians

After data cleansing, 251 data sets from veterinarians remained for further analysis, with 10% (n = 25) describing themselves as male and 90% (n = 226) as female. In terms of age, 10% (n = 25) were between 18 and 30 years old, 78.9% (n = 198) were between 31 and 60 years old, and 11.2% (n = 28) were older than 60 years (Table 3).

Just under a third obtained their degree at the Justus Liebig University Giessen, with almost another third at the University of Veterinary Medicine Hannover (Table 4).

A total of 21.1% of the participants had obtained additional qualifications. Among these, 0.8% (n = 2) were members of the European College or American Board; 13.9% (n = 35) were nationally certified veterinary specialists; 0.4% (n = 1) was nationally certified and board-certified. Furthermore, 6% (n = 15) had other additional qualifications, such as a master’s degree or veterinary qualifications awarded by the regional veterinary council.

A total of 22.3% (n =56) had been practicing small animal medicine for less than five years. 19.9% (n = 50) for five to ten years; 23.9% (n = 60) for eleven to twenty years and 33.9% (n = 85) for over twenty years (Table 3).

**Figure 1:**
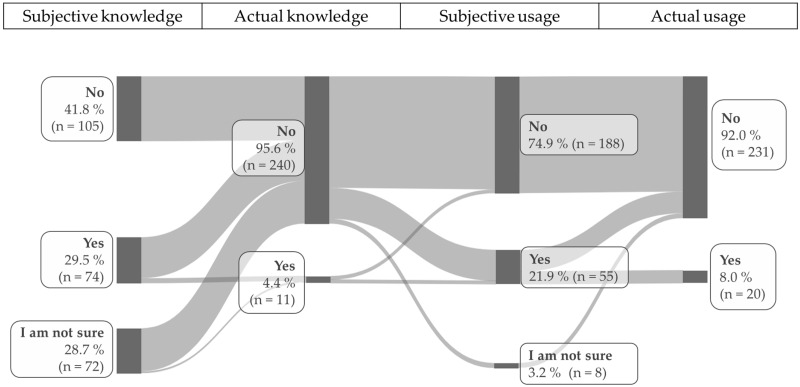
Veterinarians subjective and actual knowledge and subjective and actual usage of quality of life assessment tools.

When asked if they knew any tools to assess QoL in animals, 41.8% (n = 105) answered “no”; 29.5% (n = 74) answered “yes”; and 28.7% (n = 72) stated that they were not sure (Figure 1).

Analysis of the open-ended follow-up questions posed to those who answered “yes” or “I am not sure” yielded the following results:

Of the veterinarians who answered “yes”, 8 (10.8% of “yes” respondents; 3.2% of all veterinarians who participated in the survey) mentioned an actual tool. Furthermore, 66 (89.2% of “yes” respondents; 26.3% of all veterinarians who participated in the survey) described other approaches, such as checking vital parameters, assessing the general clinical condition, performing blood tests, lameness examination, monitoring food and water intake, and evaluating behavior in general and in more specific aspects, such as social interaction or comfort behavior (Figure 1).

Of the participants who answered “not sure,” 3 (4.2% of “I am not sure” respondents; 1.2% of all veterinarians who participated in the survey) mentioned an actual tool. Additionally, 69 veterinarians (95.8% “I am not sure” respondents; 27.5% of all veterinarians who participated in the survey) described other approaches (Figure 1).

In summary, 11 veterinarians (4.4% of all veterinarians who participated in the survey) had knowledge about specific QoL assessment tools.

After it was clarified that tools to assess QoL can include a scale or a questionnaire, participants were asked whether they use such tools in daily practice.

In total, 74.9% (n = 188) of veterinarians stated that they do not. Conversely, 21.9% (n = 55) answered “yes”. Of those, 20 (36.4% “yes” respondents; 8.0% of all veterinarians participating in the survey) mentioned actual tools, while 35 (63.6% of “yes” respondents; 13.9% of all veterinarians participating in the survey) described other approaches. Finally, 3.2% (n = 8) who stated they were “not sure” whether they use tools in daily practice, all described other approaches in the follow-up question (Figure 1).

**Figure 2:**
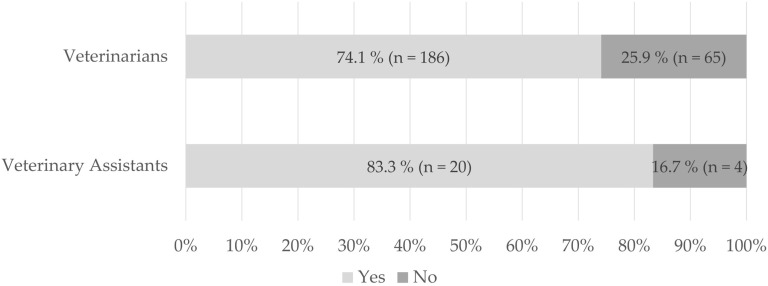
Veterinarians and veterinary assistants’ statements whether the usage of a validated quality of life assessment tool in daily veterinary practice could be possible.

When asked, if they could imagine using a validated questionnaire on a regular basis in daily veterinary practice, 74.1% (n = 186) confirmed that they could do so, while 25.9% (n = 65) denied it (Figure 2).

#### 3.1.2. Veterinarian Assistants

After data cleansing, 24 data sets from veterinarian assistants remained for further analysis, with all of them describing themselves as female. In terms of age, 54.2% (n = 13) were between 18 and 30 years old, 41.7% (n = 10) were between 31 and 60 years old, and 4.2% (n = 1) were older than 60 years (Table 3).

In terms of professional experience, 41.7% (n = 10) had worked in small animal medicine for less than five years, 25.0% (n = 6) for five to ten years, 29.2% (n = 7) for eleven to twenty years, and 4.2% (n = 1) for over twenty years (Table 3).

**Figure 3:**
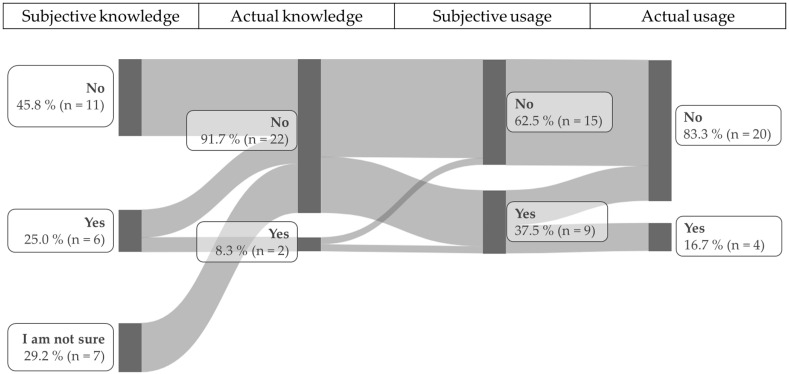
Veterinary assistants subjective and actual knowledge and subjective and actual usage of quality of life assessment tools.

When asked if they were aware of tools to assess QoL in animals, 45.8% (n = 11) answered “no”, 25.0% (n = 6) answered “yes”, and 29.2% (n = 7) stated that they were “not sure” (Figure 3).

Analysis of the open-ended follow-up questions from those who answered “yes” or “I am not sure” yielded the following results:

Of the veterinary assistants who answered “yes”, 2 (33.3% of “yes” respondents; 8.3% of all veterinarian assistants who participated in the survey) mentioned an actual tool. The remaining 4 “yes” respondents (66.7% of “yes” respondents; 16.7% of all veterinarian assistants who participated in the survey) described other approaches, such as evaluating behavior and performing laboratory tests. None of the veterinarian assistants who answered “I am not sure” mentioned an actual tool; all described other approaches (Figure 3).

After it was clarified that tools to assess QoL can include a scale or a questionnaire, participants were asked whether they use such tools in daily practice on a regular basis. Among the veterinary assistants, 62.5% (n = 15) stated they do not, while 37.5% (n = 9) answered “yes”. Of the veterinary assistants who answered “yes”, 4 (44.4% of “yes” respondents; 16.7% of all veterinarian assistants who participated in the survey) mentioned actual tools. The remaining 5 (55.6% of “yes” respondents; 20.8% of all veterinarian assistants who participated in the survey) described other approaches.

When asked if they could imagine using a validated questionnaire on a regular basis in daily veterinary practice, 83.3% (n = 20) confirmed that they could do so, while 16.7% (n = 4) denied it (Figure 2).

### 3.2. Interviews

#### 3.2.1. Knowledge of Small Animal Medicine Specialists Regarding Standardized Tools for QoL Assessment

Different levels of awareness of questionnaire-based QoL tools were found among the interviewed specialists. Some were able to name specific tools.

*“There are these quality of life scores for atopic dermatitis”*

*“There is already this FETCH questionnaire, and that is also Quality of Life, I think”*

Meanwhile, others reported only a general awareness of such tools, without being able to name specific ones.

*“There are a lot of (incomprehensible mumbling) there are different scores for Cushing’s, there are for diabetes, there are for gastrointestinal, there’s also this [name censored] app, which we helped develop, so for the gastrointestinal tract patients. Sure, I mean there are … all kinds of things”*

In some cases, knowledge existed due to past experiences.

*“We took them into account in the study by [name of PhD student]. There were a few publications, there were some from England and some from the Netherlands, which we have taken into account”*

*“Yes, of course I’m familiar with questionnaires and, as already mentioned, we also worked on this again ourselves with/so a colleague had already done this as a project”*

In other cases, it was reported that QoL instruments are taught, despite not using them in practice themselves.

*“I know they exist, I usually teach them too, but I have never used them”*

However, there are also several specialists who reported to have not yet heard of questionnaire-based QoL assessments.

#### 3.2.2. Usage of Standardized Tools for QoL Assessment by Small Animal Medicine Specialists

When asked about the use of standardized questionnaire-based QoL instruments, the interviewees described different practices. Although none reported using them in their daily veterinary routine, some described using them when doing research studies or projects.

*“I rarely need it, I have to be honest. If I do, it’s only when I’ve been involved in some kind of research; I don’t think I’ve ever used it for everyday use”*

*“if it’s not as part of a study, it’s often the case that it’s then simply included in a shortened version in the anamnesis, in the questions “how was the animal at home after the therapy?””*

*“No, for studies actually, but not in everyday practice.”*

Others mentioned that they do not assess QoL in a standardized way but sometimes assess disease severity using scores. One person also reported regularly using the Modified Glasgow Pain Scale, a validated pain scale.

*“Yes, then we have different/Well, you wouldn’t call it a questionnaire/Different scores that are used everywhere, but that’s more of a clinical assessment and not a questionnaire. These are actually the/Oh, and we have a Cushing’s questionnaire/that’s also for a score then, yes”*

*“So, we have a pain score, we use the Modified Glasgow Pain Scale in everyday practice and pain also has something to do with quality of life, but we don’t have a direct quality of life questionnaire in our practice at the moment”*

Several specialists reported using self-created tools in their daily practice, for example, to monitor chemotherapy patients. Furthermore, one participant described an attempt to create their own score system but found the process quite difficult.

*“With chemo patients, they get a control sheet, so they write it down every day: Food intake, water intake, general condition, diarrhoea, how they’re doing.”*

*“funnily enough, I actually wanted to establish a score for dogs with inflammatory brain diseases myself and wanted to involve the owners a little and took a few questions from/various validated questions from various other questionnaires. […] and I realized that the questions actually have to be asked differently for each disease, I think. And that’s why I think questionnaires are very, very difficult.”*

#### 3.2.3. Arguments for and Against the Use of Standardized Tools for QoL Assessment

When asked about their views on QoL questionnaires, participants expressed attitudes ranging from positive to neutral to negative.

*“Well-designed questionnaires are super”*

*“I think there is quite a lot of potential behind it”*

*“I have nothing against it”*

*“I hate questionnaires, but simply because I think I’m too stupid to fill them out, so whether it’s any kind of application, anything that involves ticking a box somewhere and filling it in, I’m too stupid for […] but I just don’t like doing it. I also find it awful at the doctor’s when I’m handed one of those things.”*

It was noted as a positive that such a questionnaire could strengthen communication between pet owners and their veterinarians. Furthermore, it may give pet owners the feeling that “*the veterinarian is interested in the animal*” as a whole and “*how the pet really is doing*”, which was considered extremely important for pet owners.

It was also stated that a questionnaire-based assessment was the only way to have any chance of objectifying QoL.

*“Yes, I think that makes sense, simply in order to be able to graduate”*

*“I think you have to do some kind of questionnaire to make studies and things like that more objective”*

*“You have to do it, otherwise you have nothing that can be objectified”*

*“from a scientific point of view, it certainly makes sense, you want to have something traceable and documented somehow”*

However, this very objectification is also seen as critical as participants argued that QoL is something subjective and individual. An accompanying concern was that such tools do not adequately depict the actual condition of the animal with one interviewee stating: “*if I were to adhere strictly to it, I would be afraid of missing something*”. Furthermore, participants also associated the creation of such an instrument as a difficult task with one interviewee claiming to be “*very careful when [reading] publications with such questionnaires*”.

Some interviewees did not consider the use of a questionnaire necessary for themselves, describing it as “*not essential*” and/or “*unnecessary for everyday routine*” and stated that they “*rarely need it*”.

One person associated this tool with beginners in the profession, commenting: *“To be honest, it’s something you do at university that you might also give a beginner as a guide. It’s something that, I think, once you have experience, you don’t pick up so often, but often go very specifically, very quickly in the direction you want to go.”*

In contrast another specialist described that they do not use questionnaires because they already employ a “*very structured medical history form that [they] simply always ask and write down*”.

Another person remarked that while it might be “*interesting to assess*” QoL, the information obtained is “*kind of useless*”, as long as there is no treatment approach for the issues detected.

Another reason that made some respondents sceptical about the use of questionnaires was the additional time required to implement them.

*“The question is simply how much time does a veterinarian have to do this […] with vets I always worry that they don’t have time for it”*

*“I haven’t yet found a really good grading system that is easy to implement without it costing us an infinite amount of time […] I used to do that when I was still at university. I can’t remember why we stopped, I think it was just too time-consuming”*

*“So, it’s primarily a time factor that plays a role for us rather than anything else”*

*“A vet never has time, especially not at the moment, so more than five minutes would be too many dropouts, I think, so”*

One interviewee also stated that *“there are just so many things you have to change and there’s simply not much time, a lot of stress with other things, where something like this simply falls by the wayside, to be honest”*. Another interviewee supported this view, stating that *“in everyday life [they] always make sure that [they] cut back where [they] can”*.

#### 3.2.4. QoL Assessment by Small Animal Medicine Specialists in Their Daily Veterinary Routine

The interviewees reported that a formal QoL assessment is not typically conducted in their daily veterinary routine. Instead, it is described as a general impression that forms during the entire process of taking a patient’s anamnesis (medical history), observing and examining the animal, and conversations with the owner. The amount of time dedicated to this process varies among interviewees. Some report conducting very extensive, long and comprehensive anamnesis, discussions and animal observations as one of the interviewees vividly described:

“*We take a lot of time in the anamnesis and the owners sit there and we talk and the dogs and cats are allowed to walk. And you can already see a lot there. So, I see how the connection is between them, so dog and cat with the owners, how they behave, how they walk, so this is especially with weight loading, so limbs and stuff, so it is really amazing how much you can see only by observing or especially with cats, how relaxed they walk or not when they are in the consultation. We write that down, so we simply write down what the owners tell us, but also what we observe ourselves.*”

Others reported less standardized approaches and described QoL assessment as more “*indirectly*” incorporated in questions about the animal’s general state of being:

*“I don’t ask “Has the quality of life improved?”, I ask: “Are you satisfied with the patient’s overall condition?” and then most people start talking”*

*“So, for me in a practice, “Mrs. Müller, how is he doing?” is absolutely enough. I almost always get an answer that I can do something with. And I might ask three or four more things […] so, of course, I also see things, like can the dog walk, how is he doing, is he in pain, what kind of face is he making?”*

Other interviewees stated that the assessment of QoL is done rather “*subconsciously*”, with one emphasizing that studying veterinary medicine “*has hopefully led us all to be able to evaluate this on the side, also from the anamnesis that the owner gives of themselves*”.

The extent of the documentation on QoL—or rather the overall condition of the patient since QoL as such was not formally assessed—varied among the respondents. Some reported that their documentation is quite detailed, noting that even without an explicit point concerning a QoL statement, the information is “*in principle […] clear from the entire report*”. One interviewee also stated that one “*could probably take the forms and fill them in afterwards*”.

Others reported taking notes only if there is a deviation from the normal state or “*only in strong negative cases*”. Some described documenting the overall condition in an unspecific way, such as taking notes of the phrases’ owners use: “*There are also some who say after an intervention: “He plays more, but you can still tell that he stops earlier than other dogs” and I write that down in the same way*”. Or with “*a half-sentence that says “dog is doing better” or “cat is happy” or “everything else is great”, something like that*”.

However, other interviewees stated that they do not document anything related to QoL but focus instead solely on disease-specific characteristics.

*“We are very scientific in what we note. It’s just a degree of lameness and a degree of fullness and pain. And this other/quality of life is so flexible, we don’t write it down.”*

*“We do a lot of allergy sufferers and of course they itch and with that we are dependent on the owner’s assessment and we always have a visual analogue scale that we at least imagine and say “Give a score between zero and ten” […] of course, I know there is this global assessment and this quality of life/attempt to objectify that, we don’t normally do that.”*

*“So, we have a pain score, we use the Modified Glasgow Pain Scale in everyday life and after all, pain also has something to do with quality of life”*

## 4. Discussion

This study explored the awareness, current use and willingness to use QoL assessment tools among veterinarians and veterinary assistants in Germany, using a mixed methods approach, combining an online survey, containing quantitative and qualitative elements, with qualitative semi-structured interviews.

The survey revealed that while 29.5% of veterinarians stated to have knowledge of tools to assess QoL, only 4.4% were able to reference scales, scores, or questionnaires to assess QoL or name specific tools. The remainder either reported having no knowledge of such tools or mistakenly identified other approaches, such as assessing vital parameters, as tools.

After it was explained that a tool to assess QoL can be, for example, a scale for an overall assessment or a questionnaire, 21.9% of veterinarians reported using one on a regular basis. However, when asked to describe their use of such tool only 7.9% described using scales, scores, or questionnaires; the rest described other approaches, such as checking vital parameters, assessing the general clinical condition, performing blood tests, lameness examination, monitoring food and water intake, and evaluating behavior in general and in more specific aspects, such as social interaction or comfort behavior. Notably, more veterinarians claimed to use a tool in their daily routine (7.9%), than were able to describe knowledge about such tools (4.4%). This discrepancy can be attributed to several factors. First, social desirability bias may have affected self-reported answers, meaning respondents may have wanted to appear favorable by embellishing their answers and being reluctant to admit a lack of knowledge [20]. Second, the question about the knowledge of tools was phrased openly, to avoid influencing respondents’ answers. It is therefore possible this question was too vague, preventing respondents from recalling relevant knowledge. The subsequent clarification may have then led to more precise answers regarding tool usage.

The study conducted by Roberts and colleagues [11], found that 29.1% of participants were aware of the existence of QoL tools. This figure corresponds well with the 29.5% of veterinarians in our study who stated that they were familiar with such tools but is much higher than the 4.4% in our study who could actually describe a tool. However, Roberts and colleagues [11] provided information about such tools immediately before asking participants about their awareness—a procedure that can be considered high risk for social desirability bias. Therefore, it is unclear whether the differences in reported awareness between UK and German veterinarians are genuine or the result of differently worded questions. Yet, it must be considered due to research communication occurring primarily in English, access to this information is likely easier for native English speakers than for those for whom English is a second or third language. Despite this, both studies show that only the minority of veterinarians are aware of QoL assessment tools.

Among the veterinary assistants, 8.3% (n = 2) had actual knowledge of a tool and 16.7% (n = 4) reported using one in their daily routine after the concept of such a tool was clarified. This presents the same phenomena as observed among the veterinarians, albeit with slightly higher overall knowledge and reported usage. However, due to the low sample size of the veterinary assistants (n = 24), knowledge of QoL tools among this professional group requires further investigation.

However, the majority—74.1% (n = 186) of the veterinarians and 83.3% (n = 20) of the veterinary assistants—would consider the use of a validated questionnaire to assess QoL in their daily routine.

As survey respondents were self-selected, a certain selection bias needs to be discussed: It is likely that only those completed who have at least a small interest in this topic. As a result, it has to be considered that the data presented may overestimate the general knowledge and awareness of professionals in small animal medicine.

The level of knowledge about QoL assessment tools varied among the interviewees, indicating that the dissemination of these tools within the veterinary profession is heterogenous. For a more frequent use in practice, spreading awareness of their existence should be the first step.

While assessing QoL was not done regularly by the interviewees, some had worked with such tools when involved in studies that required an objective assessment approach. Furthermore, there was experience with tools that measure QoL related constructs, such as disease severity or pain, which some interviewees used regularly. This shows that implementing questionnaires in veterinary practice is not unrealistic in principle. These findings correspond with those of Roberts and colleagues [11], who also reported higher awareness of pain assessment tools than of QoL assessment tools.

In general, the interviewees stated that they “have nothing against” the use of a QoL questionnaire and saw “quite a lot of potential behind it”. However, personal aversions to questionnaires and the associated bureaucracy were also evident. These findings show that, in addition to professional considerations, personal preferences also affect the use of QoL assessment tools.

The interviewees recognized that the use of a QoL questionnaire could strengthen communication between pet owners and their veterinarians. They noted it could give pet owners the feeling that “the veterinarian is interested in the animal” as a whole and in “how the pet really is doing”, a factor considered extremely important to pet owners. These perceptions align with the findings of Mwacalimba and colleagues [9], who also found using a QoL assessment tool deepens conversations between owners and veterinarians.

Besides recognizing that standardized assessment with a tool is the only way to objectify QoL, interviewees expressed concern that this very objectification fails to do justice to the individual and subjective nature of QoL. This highlights that such tools and their results must be handled carefully and reasonably, ensuring that clinical veterinary expertise is never disregarded. This perspective aligns with recommendations for using Patient Reported Outcome Measure (PROM) in human medicine [21].

One reason reported against implementing QoL assessment tools in daily veterinary routine was the interviewees’ perceived a lack of additional value such a tool would bring to their daily routine. Interviewees felt fully capable of assessing an animal’s condition without using a tool and therefore saw no need for one. The perception that these tools do not contribute to gathering meaningful information when a thorough anamnesis has already been carried out was also described for some professionals in human medicine [22,23].

One interviewee reported that assessing QoL information felt unhelpful where no further medical intervention was possible. This aligns with a study investigating the implementation of a PROM in dialysis centers, which also identified resistance to asking questions without being able to offer solutions [24]. In veterinary medicine, where it is possible to euthanize an animal that is suffering too much, the potential value of instruments for measuring quality of life to support end-of-life decision-making must be addressed at this point. Some attempts have been made to develop aids for end-of-life decisions from both, the veterinary [25] and the ethical [26,27] point of view and it proposes an upcoming field of research, as many ethical dilemmas are experienced in the clinical practice concerning such situations [28]. However, when developing tools to support end-of-life decisions, it is crucial to already consider the discriminative purpose of the instrument during the development process. Tools with discriminative purposes will likely differ from those with evaluative purposes, which are designed to monitor disease progression or improvements under therapy [29]. This is because the characteristics required for good discriminative purposes partly contradict to those required for good evaluative purposes and the other way round [30].

Another reason against the regular use of QoL assessment tools was the perceived lack of time in daily practice. This perceived shortage of time has been identified as a barrier to implementing QoL tools in other studies as well [11]. This sentiment was echoed by two interviewees who stated that there are so many things to do and implement that they have to “cut back where [they] can”.

This statement reflects not only the perception that the QoL tool is not beneficial enough to justify the time investment, but also the broader issue of veterinarians being overworked and under high levels of stress [31,32].

However, it should be noted that an evaluation of a QoL assessment tool in two clinical settings found that its use required no additional time [9].

While the interviewees did not perform a specific (QoL) assessment, their approach to animal assessment varied in scope. Some reported conducting a detailed anamnesis and observation with detailed documentation, while others described more targeted conversations. QoL was therefore mostly assessed subconsciously and indirectly by assessing the animal‘s overall state and behavior and including the results of disease-specific examinations. The survey respondents also reported the use of clinical data, such as vital signs, overall condition and behavior to assess QoL. The findings from the survey therefore correspond well with those obtained from the interviews and align with the fact that the term QoL in veterinary medicine is closely linked with the concept of health and has long been used interchangeably with the extent of clinical symptoms [33]. However, it encompasses more than health, including emotional and social aspects among others [28,34,35,36], and the consideration of general and specific behaviors by some respondents indicate, that these aspects are taken into account by those respondents.

If documented at all, specific notes on QoL were mostly made only when it deviated in a strongly negative way. This focus on the deviation from a normal state or the dysfunction of biological processes is well established in veterinary medicine [37,38].

In human medicine, there is discussion about implementing a more person-centered approach within the recently more diagnosis-centered framework [39]. Similarly, in veterinary medicine, there are calls to rethink the conceptualization of health to prospectively include animal welfare, moving beyond just disease treatment [40].

The findings of the interviews therefore align very well with the results of the survey. Both show a limited awareness of such tools and little to no usage of such tools in daily routine among veterinarians in Germany.

## 5. Conclusions

In summary, while knowledge of existing QoL tools is inconsistent among veterinarians and their use is mainly confined to participation in studies, a majority of veterinary professionals can imagine regularly using a validated QoL tool.

Qualitative interviews revealed that concerns include the perceived lack of recognition of QoL’s individuality and subjectivity, as well as the lack of time to familiarize themselves with, apply, evaluate, and act upon a questionnaire, especially if it reveals problems with no clear medical solution.

Simultaneously, veterinarians recognized the benefits of standardized QoL assessments and recognized their potential to strengthen professional-client bonds. They acknowledged that pet owners highly value the perception that the veterinarian is interested in the animal as a whole.

Therefore, researchers should not only focus on reliability and validity when developing tools but also critically examine usability. Moreover, such tools should indeed come with clear instructions and be widely promoted within the framework of scientific communication so that they can be readily adopted. The time constraints and cognitive load of practitioners must be considered by designing tools that are as user-friendly as possible. Ensuring easy accessibility, potentially through electronic questionnaires that can be integrated directly into patient records and provide automated preliminary evaluations, could significantly facilitate implementation. This would support a holistic, patient-centered approach and complement the currently predominant, subconscious method of forming an impression.

## Figures and Tables

**Table 1:** Areas of specialty of the interviewees.

Area of Specialty	Number of Specialists Interviewed	Case Number
Orthopaedics	4	6, 13, 14, 15
Oncology	3	5, 7, 11
Neurology	3	2, 8, 12
Internal Medicine	3	1, 3, 10
Cardiology	2	4, 9
Dermatology	1	16

**Table 2:** Main categories of the qualitative content analysis and their category definition.

Main Category	Category Definition
Knowledge about/awareness of QoL assessment tools	All statements that contain specific or vague knowledge or awareness about questionnaires for assessing quality of life and statements that contain a corresponding lack of knowledge.
Use of QoL assessment tools	All statements on the use and non-use of questionnaires to assess quality of life.
Attitude towards questionnaire-based QoL assessment tools	All statements on the use of questionnaires in general or on personal attitudes toward the use of questionnaires.
Own practice of assessing QoL of their patients	All statements regarding the assessment and/or documentation of a patient’s quality of life in one’s own veterinary practice and statements regarding the absence of such assessment and/or documentation.

**Table 3:** Demographic data for the participating veterinarians and veterinary assistants.

	Veterinarians	Veterinary Assistants
**Gender**		
Female	90% (n = 226)	100% (n = 24)
Male	10% (n = 25)	-
**Age**		
18–30 years	10% (n = 25)	54.2% (n = 13)
31–60 years	78.9% (n = 198)	41.7% (n = 10)
>60 years	11.2% (n = 28)	4.2% (n = 1)
**Experience in small animal medicine**		
<5 years	22.3% (n =56)	41.7% (n = 10)
5–10 years	19.9% (n = 50)	25.0% (n = 6)
11–20 years	23.9% (n = 60)	29.2% (n = 7)
>20 years	33.9% (n = 85)	4.2% (n = 1)

**Table 4:** Universities where the participants obtained their degrees.

University	Number	Percentage
Freie Universität Berlin	40	15.9
Humboldt-Universität zu Berlin *	3	1.2
University of Veterinary Medicine Hannover	68	27.1
Justus Liebig University Giessen	73	29.1
Ludwig Maximilian University of Munich	32	12.7
Leipzig University	25	10.0
Degree obtained abroad	10	4.0
Total	251	100.0

* At the time of the division of Germany, it was also possible to study veterinary medicine at the Humboldt University in Berlin. As this was only limited to the time of the division, the numbers are correspondingly low. Today it is no longer a place to study veterinary medicine in Germany.

## Data Availability

The data presented in this study are available on request from the corresponding author due to legal reasons.

## Supplementary Materials

The following supporting information can be downloaded at: https://www.mdpi.com/article/10.3390/ani15243617/s1, Figure S1: Excerpt and sequence of relevant questions from the survey; Table S1: Excerpt of relevant questions from the interview guide.

## Abbreviations

The following abbreviations are used in this manuscript:

QoL	Quality of life
PROM	Patient reported outcome measure
