# Supplementary material for: Assessment of the Awareness and Use of Quality of Life Tools in Small Animal Practices in Germany
Source: Animals (Basel). 2025 Dec 16;15(24):3617. doi: 10.3390/ani15243617 (PMC12730036; doi:10.3390/ani15243617)
Supplement: Supplementary file 1 [file animals-15-03617-s001.zip › animals-4008424-supplementary-Table S1.pdf]

Table S1. Excerpt of relevant questions from the interview guideline

9.	How do you assess an animal's quality of life in your daily practice?
10.	What is your general opinion of the use of tools, like questionnaires, in assessing the quality of life of an animal?
11.	Do you know any concrete tools to assess quality of life?
12.	Do you use tools or questionnaires in everyday life?
13.	Is the quality of life of patients documented in any way in everyday life?
