# Supplementary figures and images for: Assessment of the Awareness and Use of Quality of Life Tools in Small Animal Practices in Germany
Source: Animals (Basel). 2025 Dec 16;15(24):3617. doi: 10.3390/ani15243617 (PMC12730036; doi:10.3390/ani15243617)

**Figure S1.** Excerpt and sequence of relevant questions from the survey

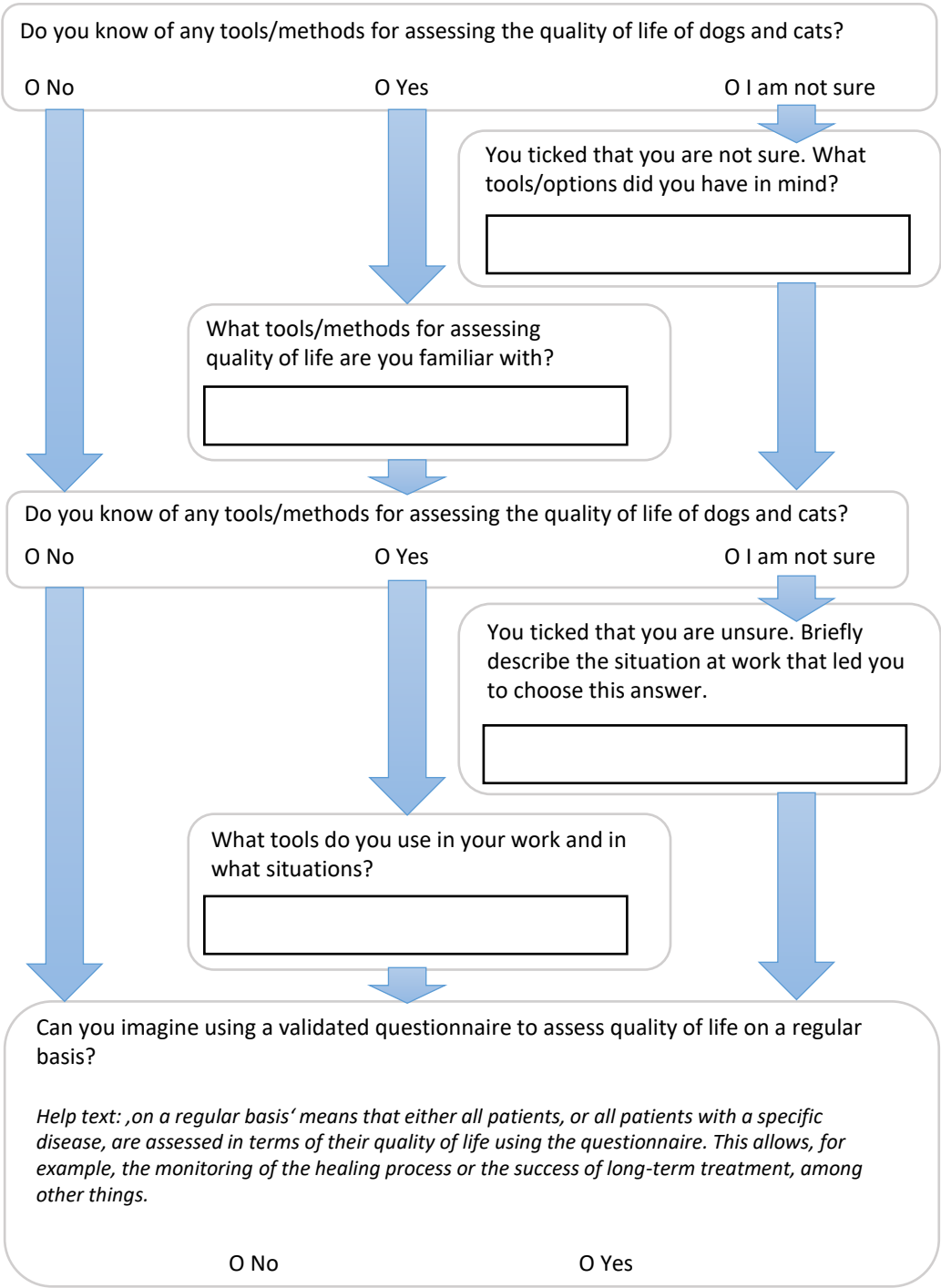

Supplement: Supplementary file 1 [file animals-15-03617-s001.zip › animals-4008424-supplementary-Figure S1.pdf]
